# One Health Perspectives on Food Safety in Minimally Processed Vegetables and Fruits: From Farm to Fork

**DOI:** 10.3390/microorganisms11122990

**Published:** 2023-12-15

**Authors:** Maria Isabel Santos, Madalena Grácio, Mariana Camoesas Silva, Laurentina Pedroso, Ana Lima

**Affiliations:** 1 Faculty of Veterinary Medicine, Lusófona University, 1749-024 Lisbon, Portugal; mrcamoesas19@gmail.com (M.C.S.); laurentina.pedroso@ulusofona.pt (L.P.); 2 CECAV—Centre of Animal and Veterinary Science, Faculty of Veterinary Medicine, Lusófona University, 1749-024 Lisbon, Portugal; 3 Instituto Superior de Agronomia, University of Lisbon, 1349-017 Lisbon, Portugal; madalenagracio@outlook.com

**Keywords:** minimally processed foods, foodborne pathogens, One Health, fruits and vegetables food chain production

## Abstract

While food markets and food production chains are experiencing exponential growth, global attention to food safety is steadily increasing. This is particularly crucial for ready-to-eat products such as fresh-cut salads and fruits, as these items are consumed raw without prior heat treatment, making the presence of pathogenic microorganisms quite frequent. Moreover, many studies on foodborne illnesses associated with these foods often overlook the transmission links from the initial contamination source. The prevention and control of the dissemination of foodborne pathogens should be approached holistically, involving agricultural production, processing, transport, food production, and extending to final consumption, all while adopting a One Health perspective. In this context, our objective is to compile available information on the challenges related to microbiological contamination in minimally handled fruits and vegetables. This includes major reported outbreaks, specific bacterial strains, and associated statistics throughout the production chain. We address the sources of contamination at each stage, along with issues related to food manipulation and disinfection. Additionally, we provide potential solutions to promote a healthier approach to fresh-cut fruits and vegetables. This information will be valuable for both researchers and food producers, particularly those focused on ensuring food safety and quality.

## 1. Introduction

Modern societies have undergone profound economic, social, demographic, cultural, and food changes. Currently, there is a relative abundance of food for most populations [1]. However, this scenario is evolving. After years of decline, various forms of hunger and malnutrition, including obesity and micronutrient deficiencies, are on the rise [1,2,3,4,5]. Adding to this, the fast pace of modern life and demanding work hours result in less time for meal preparation. This leads consumers to prefer foods that are not only healthy and easy to prepare [6,7,8,9,10] but also of high quality and safety, preferably without additives [7,9,11]. A wide range of ready-to-eat, refrigerated food products with longer shelf lives is available to meet these preferences [10,11,12,13,14]. Among these, a specific category of products known as minimally processed foods (MP) [7,15,16] has emerged. These include fresh-cut vegetables, meat, and fish, marketed and packaged for immediate consumption for ease and convenience [17,18,19].

Being fresh and minimally processed and consumed raw, food safety in MP products has become a top priority for both public and private sectors. Despite global efforts in risk assessment, traceability, hygiene, hazard analysis critical control points (HACCP), and the withdrawal of unsafe products from the market, food safety faces unprecedented challenges today. Pathogen contamination is responsible for a wide variety of diseases, ranging from diarrhea to cancer [20,21]. Approximately 600 million people worldwide fall ill due to contaminated food, with 420,000 deaths annually, resulting in the loss of 33 million disability-adjusted life years [22]. Children younger than 5 years old bear 40% of the total foodborne diseases, accounting for 125,000 deaths every year. Diarrheal diseases, causing 550 million illnesses and 230,000 deaths annually, are the most common illnesses resulting from the consumption of contaminated food [21]. According to the 2021 EFSA/ECDC Report in Europe [23], the causative agents most frequently identified in foodborne outbreaks are *Salmonella*, bacterial toxins, norovirus, *Listeria monocytogenes*, *Campylobacter*, and Shiga toxin-producing *E. coli*, with *L. monocytogenes* being responsible for most deaths. All of these can occur in MP produce.

An important consideration in foodborne outbreaks, particularly in MP fruits and vegetables, is that they are often investigated at the end of the food chain production. However, a more comprehensive approach involves investigating across human, animal, and environmental sectors, each informing the other synergistically. It is widely recognized worldwide that achievements in the areas of food safety and public health will only be realized through a One Health approach, integrating, and sharing information on environmental, animal, and human health, recognizing their interconnectedness and importance in efficient health systems [24,25]. Despite efforts towards One Health strategies, food safety in MP fruits and vegetables has been largely neglected. In fact, there is a lack of reviews that encompass the overall picture of vegetable and fruit contamination, from the farm environment to the consumers, concerning foodborne diseases. In this context, our goal was to review information concerning microbiological food contamination sources in minimally processed fresh foods, encompassing each step and stage in the production chain from the producer to the final consumer and accounting for major outbreaks and strains.

## 2. Minimally Processed Fruits and Vegetables

It is widely recognized that fruits and vegetables play an essential role in maintaining a healthy diet. Fruits and vegetables serve as vital sources of nutrients an provide extensive health benefits [4,7,14,16,26,27,28]. Insufficient intake of fruits and vegetables is estimated to contribute to 14% of worldwide gastrointestinal cancer deaths, 11% of deaths attributable to ischemic heart disease, and 9% of deaths caused by stroke [29]. Currently, a daily consumption of 400 g of fruits and vegetables, equivalent to five individual portions, is recommended [30,31,32,33]. In Europe, as of 2021, the median intake of fruits and vegetables was 364.58 g per day per capita, reflecting a 1.27% increase compared to the previous five years’ median [34]. After the United Nations General Assembly declared 2021 as the International Year of Fruits and Vegetables [35], minimally processed fruits and vegetables (MPFV) have gained popularity among consumers. MPFV refers to any fresh vegetable or fruit that has undergone physical alterations from its original form, then is packaged, but remains in a fresh and ready-to-use state [7,36]. According to Parrish [37], the nutritional characteristics of most of these products remain similar to those of the original product whilst simplifying meal preparation, saving time, and reducing waste. Indeed, throughout the last decades, the market for MPFV has been expanding, with global sales reaching 72.61 million tons in 2017, generating revenue of approximately USD 48 billion and experiencing a 5.7% increase [38].

### 2.1. Minimally Processed Fruit and Vegetable Contamination

Minimally processed fruits and vegetables (MPFV) are not sterile products; instead, they undergo only a moderate decrease in the microbiota present during processing. As vegetables are raw agricultural products, MPFV will likely contain potentially pathogenic microorganisms [26,39,40,41,42,43,44,45,46,47] that can arise from different steps in the food chain production. It is, therefore, unsurprising that some of the most nutritionally recommended foods pose significant challenges in terms of food preservation and safety. In recent years, foodborne outbreaks associated with the consumption of raw fruits and vegetables have been on the rise, prompting increased attention from researchers and health authorities to investigate food safety aspects related to microbial contamination of fresh produce [4,45,47,48,49,50,51,52]. Table 1 lists the key factors contributing to the emergence of fresh produce as a source of foodborne outbreaks. The heightened risk is partly attributed to the intensive and centralized nature of preparations and distributions, increasing the likelihood of incorrect handling and/or storage practices. Additionally, the absence of heat treatment during the food chain production further complicates efforts to eliminate the risks associated with the consumption of uncooked vegetables [6,16,45,47,53,54,55].

### 2.2. Main Sources of Microbiological Contamination throughout the Food Chain Production

**Figure 1:**
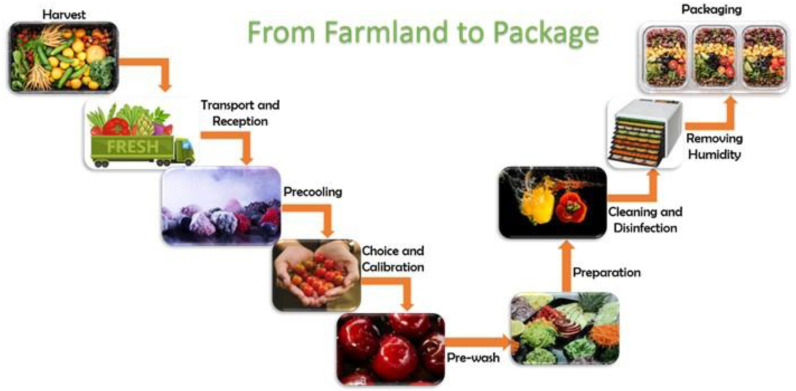
Phases of minimally processed fruit and vegetable processing, from the farmland to packaging. Adapted from [39,40,41,42,43].

Contamination of vegetables can occur at various stages: in the pre-harvest phase while the plant is in the field, during harvesting, and in the post-harvest phase, encompassing transport, processing, or packaging [28,45,46,51,57]. The different stages of salad processing involve several steps, as observed in Figure 1:

Typically, initial contamination in the plant reflects its environmental microbiota [51,56]. This microbiota comprises microorganisms responsible for product alteration, many of which reside on the plants throughout their life cycle. While these naturally occurring microorganisms are generally non-pathogenic bacteria, the fact that these products are cultivated in natural environments makes them susceptible to contamination by pathogenic microorganisms with health implications [4,46,58,59]. Therefore, it is crucial to develop strategies to identify the sources of pathogenic microorganisms at each stage of the food chain production process to prevent the contamination of plants throughout [47,57].

#### 2.2.1. Soil

Pathogenic microorganisms of enteric origin can persist for extended periods in human and animal feces, posing a risk of contaminating land and crops [53,60,61,62,63,64,65]. Notably, *Escherichia coli* O157:H7 can endure for over seven months in soils exposed to rainy winter conditions. Furthermore, the widespread use of inadequately composted manure or feces from domestic or wild animals to fertilize and enhance soil structure has contributed to the spread of these microorganisms in the environment [57,63,66]. Crops, especially those growing closer to the ground, such as lettuce, are particularly vulnerable, as they may encounter soil during cultivation, irrigation, or heavy rains [65,67,68]. To mitigate the risk of introducing pathogens through organic fertilizer, it is recommended to allow a minimum of 90–120 days to pass (depending on whether the edible portions are in contact with the soil or not) between manure application and plant harvesting. The risk of pathogen presence decreases as the time between manure application and produce harvest increases [66,68,69].

#### 2.2.2. Irrigation Water

Another significant contamination source is the use of contaminated water for irrigation or the application of pesticides. Foodborne outbreak investigations by the Centers for Disease Control and Prevention (CDC) have linked irrigation water to pathogen contamination of produce [70,71,72]. Studies by Dobhal et al. [73] have shown that strains of *Salmonella Typhimurium* and *E. coli* O157:H7 can survive in pesticide solutions. This situation is more prevalent in regions with water scarcity or where effluents are utilized for irrigation.

As natural reservoirs of *E. coli* and *Salmonella* include cattle, goats, and sheep, the intensification of animal production contributes to increased environmental and water contamination through runoff from production areas. The unrestricted access of farm or wild animals to cultivated fields or irrigation water is another important factor, as they can carry strains of Shiga toxin-producing *E. coli* (STEC) and other pathogenic microorganisms [46,66,67,74]. Several foodborne outbreaks resulting from produce contamination through irrigation water have been reported. For instance, in Sweden in 2015, an *E. coli* O157 outbreak was linked to contaminated water from a river used for irrigation of lettuce. Similarly, in the United States in 2008, a large outbreak caused by the consumption of serrano and jalapeño peppers was associated with contaminated irrigation water. Another incident occurred in 2010 when alfalfa sprouts were contaminated by *Salmonella*, and water runoff revealed the presence of the outbreak strain. In 2015, a multistate outbreak occurred due to the consumption of tomatoes irrigated with contaminated water [75]. Machado-Moreira et al. [51] reported *E. coli* and *L. innocua* contamination in lettuce due to spray irrigation with contaminated water, while Coleman et al. [76] demonstrated the contamination of hydroponic tomato plants grown with nutrient solution contaminated with *Salmonella enterica*.

The situation is particularly concerning due to variations in water regimes observed in recent years, including seasonal floods leading to fecal contamination and subsequent crop contamination. Conversely, dry summers have resulted in an increased reliance on wastewater, derived from effluent treatment on farms, to irrigate vegetable crops. As *E. coli* and *Salmonella* spp. can survive well in sediments, seasonal flooding during rainy seasons further contributes to increased contamination [77,78]. The use of untreated human sewage can also be a source of various pathogens, including Shigella spp., *Salmonella enterica*, different *E. coli* pathotypes, and enteric viruses [70,78,79]. Additionally, natural disasters such as fires and seasonal floods can lead to fecal contamination and subsequent produce contamination [24].

#### 2.2.3. Insects

Insects are also a source of contamination for crops [46,53]. Flies are attracted to manure and can carry and transmit pathogenic microorganisms [80]. Experiments with the fruit fly (*Ceratitis capitata*) contaminated with *E. coli* strain labeled with a fluorescent protein have demonstrated that this insect can transmit pathogenic bacteria to intact fruits [81]. In addition to transmitting pathogenic microorganisms, insects can damage plant tissues by destroying the waxy cuticle, which is the first defense barrier, making them more vulnerable to pathogen penetration [80].

#### 2.2.4. Human Manipulation

During harvesting, the contamination of products is exacerbated by poor hygiene practices among rural workers and the lack of sanitation facilities [40,57,64,68,78]. In the post-harvest phase, various factors can contribute to contamination. These include the use of contaminated ice or water, inadequate hygiene practices by handling staff or consumers, damage to plant tissues, issues with transport equipment, the presence of animals or pests in the environment, water quality during production and processing, use of contaminated equipment, cross-contamination, and improper storage conditions [46,64,82,83].

**Figure 2:**
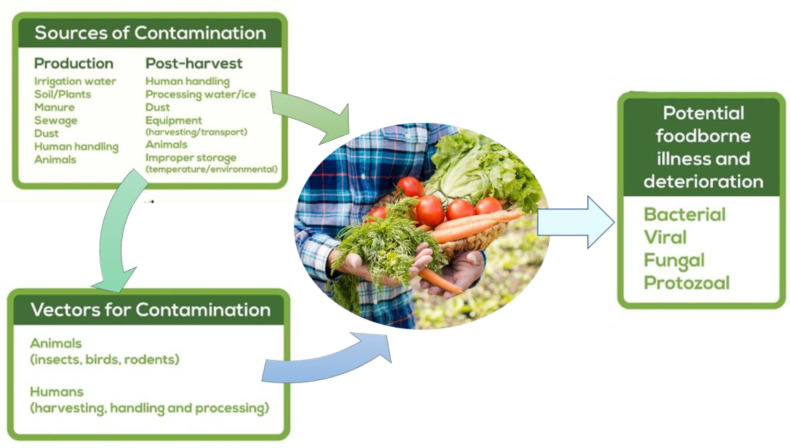
Sources of contamination in vegetables crops during production and post-harvest; source: adapted from [87].

Concerning ready-to-eat vegetables, the primary source of contamination during the processing of lettuce and other minimally processed (MP) vegetables is the cutting stage. This operation has drawbacks, as it is during this stage that vegetables are most susceptible to mechanical damage. Moreover, an increase in the specific surface area makes tissues less effective barriers to the penetration of microorganisms. This results in a loss of cellular integrity, leading to physiological changes when the substrates encounter endogenous enzymes, rapid enzymatic catalysis reactions, and the growth of harmful bacteria [4,7,40,64,84,85,86] (Figure 2).

### 2.3. Main Types of Contamination

Overall, when examining the primary types of contamination in minimally processed fruits and vegetables (MPFV), it is essential to account for the existence of a normal and natural microbiota in plant tissues that may be present at the time of consumption. The key considerations involve contamination by microorganisms responsible for product deterioration and those accountable for causing foodborne diseases. These categories encompass, respectively, microbial quality indicators linked to decay-inducing microbiota and the pathogenic microorganisms themselves.

#### 2.3.1. Microbial Quality Indicators

Being simple tests to perform, the detection and counting of aerobic mesophilic or psychotropic microorganisms and of *Enterobacteriaceae* and coliforms have been the most widely used by the minimally processed fruits and vegetables (MPFV) industry as hygiene and quality indicators. This is done to compare the mesophilic or psychotropic microorganisms’ counts in MPFV at the time they are processed with those that are present in the natural product [7,78]. MPFV are referred to as more susceptible to microbial multiplication than unprocessed products due to the presence of cutting surfaces, increased nutrients available, plant tissue respiration, and confinement within the package. Additionally, there are no treatments to ensure microbiological stability [88,89]. Several studies report that the mesophilic or psychotropic levels present in lettuce or packaged salads vary widely between 3.0 and 9.40 log cfu.g^−1^ (Table 2).

Most of the bacteria identified in raw vegetables belong to the group of rod-shaped Gram-negative bacteria (80 to 90%) and include *Pseudomonas* spp., *Flavobacterium* spp., *Enterobacter* spp., *Alcaligenes* spp., *Xantomonas* spp., *Klebsiella* spp., *Serratia* spp., and *Chromobacterium* spp. The family *Enterobacteriaceae*, which includes the coliform group, constitutes about 10% of the microorganisms found in the total enumerations [89,98]. Poubol and Izumi [99], in mango cubes preserved in a CO_2_ atmosphere, reported that the predominant microbiota was Gram-negative rod-shaped bacteria, of which 60% were *Enterobacteriaceae*. Thus, high levels of these microorganisms are habitual in minimally processed fruits and vegetables (MPFV) and are not indicative of fecal contamination; however, they may compromise their sensorial and nutritional quality [100].

Currently, *E. coli* is considered the best marker of fecal contamination [101,102,103,104], and many published studies report low prevalence as well as low levels of this microorganism. Table 3 aims to emphasize the above in the light of these indicators. It presents results reported in the scientific literature related to *Enterobacteriaceae* and coliform enumeration and the percentage of *E. coli*-positive samples, reinforcing that, in general, the levels detected were low. In a recent study carried out in Norway by the Norwegian Food Safety Authority on ready-to-eat lettuce and sugar snap peas in 2021, in a total of 118 samples, of which 37 were sugar peas and 81 MP and ready-to-eat leafy greens, *E. coli* was found in 11 samples (10 samples of lettuce and only 1 sample of sugar peas) but in low levels. Only one sample of lettuce exceeded 2 log cfu.g^−1^, the reference value indicated in Regulation (EC) N° 2073 [105].

#### 2.3.2. Pathogenic Microorganisms and Foodborne Disease-Related Cases

As previously mentioned, in addition to plant deteriorating microorganisms, it is crucial to consider pathogenic microorganisms transmitted by the produce as well. Over the last thirty years, the epidemiology of infectious diseases originating from food has undergone a significant shift, with plant products emerging as new vehicles for the transmission of zoonotic agents [51,70,106,107]. The scientific literature documents numerous outbreaks of this nature, some resulting in the tragic loss of hundreds of lives [7,48,53,70,89,107,108]. *Salmonella* spp., *E. coli* O157:H7, and *L. monocytogenes* are identified as the primary pathogenic microorganisms causing the most concern in such outbreaks [48,70,107,109]. A study conducted in Norway did not detect *Salmonella* spp. in any of the 118 samples [105]. In Europe, according to the European Union One Health 2021 Zoonoses Report [23], the frequency of these pathogens in ready-to-eat vegetables and fruits in 2021 is relatively low, as illustrated in Table 4.

Regarding outbreaks in 2021, vegetables, fruit juices, and related food products were responsible for 9.6% (*n* = 34) of confirmed outbreaks with strong evidence, while fruits, berries, and juices and their products accounted for 0.60% (*n* = 2) of such outbreaks, representing a significant increase, more than twice compared to 2020. It is noteworthy that a diverse range of causative agents were involved, including several *Salmonella* serovars implicated in 11 outbreaks, Shiga toxin-producing *E. coli* (STEC), Enteroinvasive *E. coli* (EIEC), Enterotoxigenic *E. coli* (ETEC), *Yersinia enterocolitica*, bacterial toxins such as *Staphylococcus aureus*, *Clostridium botulinum, Bacillus cereus*, unspecified bacterial toxins, viruses including *Norovirus*, and *Cryptosporidium parvum*. A total of 1715 individuals fell ill, with 131 hospitalizations, although there were no reported deaths. In addition to the three mentioned pathogens, other less frequent agents were also observed, impacting the safety of plant products and consequently the health of consumers. It is important to note that the average size of outbreaks attributed to this type of food (50 cases/outbreak) was significantly higher than those occurring due to the consumption of animal-origin foods (11 cases/outbreak) [23]. Among these occurrences, a notable outbreak was linked to Galia from Honduras. The implicated pathogen was *Salmonella Braenderup* sequence type 22, responsible for 348 illnesses and 68 hospitalizations between March and July 2021 in 12 European countries, including the United Kingdom.

It is worth mentioning that since 2012, Hepatitis A virus (HAV) outbreaks have been a recurrent problem in Europe, associated with frozen berries’ consumption. In June 2018, in Sweden, an HAV outbreak occurred linked to frozen strawberries imported from Poland. In October of the same year, in Austria, an HAV outbreak with a strain with the same genotype as the Sweden strain was also reported. The study has also concluded that the strawberries were acquired from the same producer from Poland [110]. Later, in Germany, from October 2018 until January 2020, the same HAV strain was responsible for 65 cases of the illness in 2 peaks (August to December 2018 and June to September 2019). The epidemiological research allowed us to conclude that frozen strawberry cakes were the implicated food vehicle in both outbreak waves. The traceback investigations and phylogenetic analyses have demonstrated the strain (the same identified in Sweden and Austria outbreaks and the Polish producer) was also found in berries, sewage, and stools in Egypt, raising the hypothesis that the contamination occurred in this country. As the producer from Poland has received strawberries from Egypt through a German distributor, a unique contaminated batch may have caused all referred outbreaks [111].

Another major outbreak occurred between May and July 2011, marking a significant historical reference due to an unusually high number of cases and the challenges in finding the source of infection. This outbreak took place in Germany, resulting in 3816 cases, of which 845 developed hemolytic uremic syndrome (HUS) and 54 fatalities were reported. Notably, a majority (88%) of HUS cases were observed in adults, contrasting with typical infections by VTEC strains that usually affect children. Moreover, females, particularly those aged between 30 and 34 years, were the most affected, constituting 68% of HUS cases and 58% of gastroenteritis cases. The epidemic strain was identified as *E. coli* O104:H4 enteroaggregative, which had acquired the stx2a conversion bacteriophage. This outbreak gained international attention, with cases reported in 15 other countries in Europe and the USA. In France, eight cases were reported in individuals who attended a community event, and the isolated strain in these patients was genetically compatible with the epidemic strain from Germany. The investigations traced the outbreak back to the consumption of fenugreek sprouts, with the seeds of the implicated batch imported from Egypt in 2009 [112,113,114].

**Figure 3:**
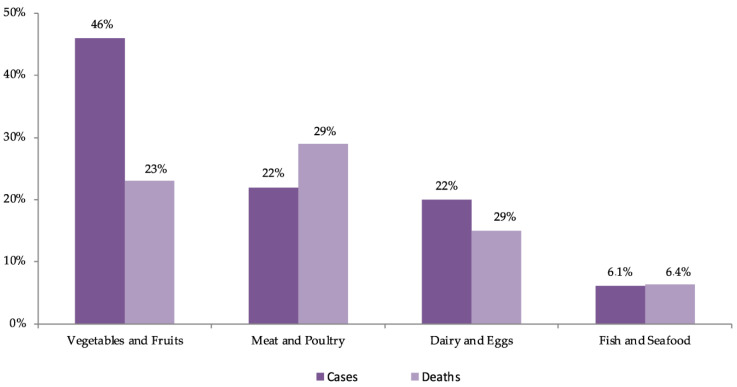
Distribution (%) of estimated cases of foodborne illness and deaths by food category in the USA in the period 1998–2008; source: [48,115].

As illustrated in Figure 3, data from the USA estimate that between 1998 and 2008, fruits and vegetables were responsible for 46% of outbreaks, mostly caused by norovirus, *Salmonella* spp., and *E. coli* O157:H7, with leafy vegetables being the most frequent vehicle. Leafy vegetables were responsible for 2.2 million cases per year, representing 22% of all cases and constituting the food product responsible for the largest number of patients. Approximately 24,000 people (41%) are hospitalized each year due to the consumption of plant-origin products, with 38% attributed to fruits and vegetables and 16% to leafy vegetables, making dairy products the leading cause of hospitalizations. In terms of the number of deaths, fruits and vegetables account for 333 cases per year (23%), considerably lower than the 43% attributed to the consumption of animal products (terrestrial). In summary, leafy vegetables account for the largest number of patients (22%), making them the second most frequent cause of hospitalizations (14%) and the fifth cause of death (6%) [48].

However, in the USA, between September 2013 and May 2016, an outbreak caused by *L. monocytogenes*, associated with frozen vegetables consumption, occurred, which affected only nine persons (all were hospitalized), and three of them have died, which corresponds to 33.3%, and this percentage is superior to that reported in the literature [116]. The outbreaks occurred, particularly, in 2011 caused by *E. coli* O104:H4 in vegetable sprouts, in 2006 by contamination of spinach MP with *E. coli* O157:H7, in 1996 also with *E. coli* O157:H7 in lettuce, and *Salmonella* spp. in tomato, juice, fruits, and sprouts reinforce the concern with products that are consumed raw and calls attention to the need to increase preventive strategies [108,114,117,118].

A study conducted by the Interagency Food Safety Analytics Collaboration, utilizing data from 1998 through 2020, estimated the percentage of illnesses caused by three priority pathogens—*Salmonella* spp., *E. coli* O157, and *L. monocytogenes*—in the year 2020. Unfortunately, no results were provided for the fourth priority pathogen, *Campylobacter*. The study, based on 1287 outbreaks, indicated that 960 were caused by or suspected to be caused by *Salmonella* spp., 272 by *E. coli* O157, and 55 by *L. monocytogenes*. Regarding produce, the conclusions are summarized in Table 5, highlighting that for *E. coli* O157 and *L. monocytogenes*, the estimated attribution percentage of produce involved in foodborne diseases is quite high [119].

According to the CDC, several foodborne outbreaks linked to produce continue to occur. A summary of the outbreaks that occurred in the last five years, and which are closed, can be seen in Table 6. Regarding the total number of illness cases, there is a percentage of deaths of 0.26%: 0.11% due to *E. coli* O157:H7 and 0.15% to *L. monocytogenes*. However, if it considered the total number of cases linked to *E. coli* O157:H7, the case fatality is 2.7%, while for *L. monocytogenes* it is 12.5% [120].

Yet another pathogenic microorganism that has raised some questions about its possible transmission through the consumption of produce is *Clostridioides difficile* (previously known as *Clostridium difficile*) [121]. This is an anaerobic spore-forming pathogenic bacteria that acts negatively in the gastrointestinal tract, causing a serious illness, particularly in hospitalized persons, subject to prolonged treatment with antibiotics. However, in the last two decades the incidence of *C. difficile* infection (CDI) has increased in the community, oftentimes in people with no history of hospitalization or antibiotic treatment [122,123,124,125]. The rapid expansion of community-acquired CDI raised the hypothesis that *C. difficile* present in the environment, animals, and retail foods causes this infection in humans [123,125,126,127]. In addition, several studies demonstrate the presence of *C. difficile* in several foods, including meat, ready-to-eat salads, and raw vegetables (such as cucumber, onions, carrots, etc.) [128,129,130,131]. Given that *C. difficile* is present in water, animal feces, and livestock manure compost, it could easily be transferred to vegetables. Table 7 presents the results from several studies conducted in diverse countries, and the positive percentage is relatively low everywhere, ranging between 2.4% and 10%. However, based on the published works, it can be inferred that the overall population is exposed to low levels of *C. difficile* through vegetable consumption. As of now, no outbreaks have been linked to foods contaminated with this pathogen, so it is not possible to classify it as a foodborne pathogen.

### 2.4. Major Decontamination Methodologies: Related Problems and Possible Solutions 

It is crucial to emphasize that MPFV do not undergo any stage ensuring the elimination of the risk related to their consumption, as they lack heat treatment to eliminate pathogens, spores, and toxins at safety levels. Therefore, the vegetable sanitation/disinfection stage, especially in MPFV, becomes critical for food safety. MPFV plants are cut, and the damage to cells makes them more susceptible to microbiological multiplication and biochemical alterations, intensifying respiratory rates and enzymatic activity [26,43,46,89,134,135,136,137].

While washing and disinfection steps of plant products are moderately effective, they are by no means efficient when dealing with internalized pathogenic microorganisms. Pathogens can penetrate plant tissues either in the pre-harvest phase through internalization or in the post-harvest phase through infiltration, complicating the situation. Infiltration, or the suction effect, can occur when a product at room temperature is submerged in colder water, creating a vacuum that sucks water and, if present, pathogenic microorganisms into the tissues through pores, channels, or fissures [15,74,137,138,139,140,141]. Studies have shown that tomatoes submerged in an *E. coli* O157:H7 suspension exhibited contamination, and *Salmonella* Typhimurium was found to infiltrate baby spinach during washing operations, dependent on humidity, temperature, and illumination conditions [142,143]. Internalization may also occur during flowering, with pathogenic microorganisms entering through flowers carried by water or insects, through roots from contaminated soil or water, through wounds or cracks, or by entrapment in the waxy film [144]. Laboratory studies have demonstrated the entry of pathogens into plant tissues through natural openings such as stomata, root, and flower junctions, or through tissue damage. After binding to plant tissues, microorganisms have the capability to form biofilms, thereby improving their capacity to endure within the plant structure [74,145,146]. In essence, the internalization of pathogenic microorganisms can take place at any point in the plant’s life cycle, progressing to subsequent phases such as seed, germination, mature plant, flower, and fruit [52].

**Figure 4:**
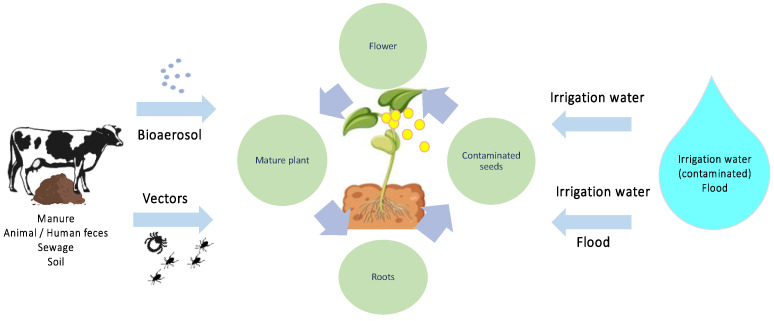
Schematic representation of the entry and permanence of pathogenic microorganisms in the plant life cycle. Adapted from: [52].

Figure 4 aims to demonstrate the entry points of pathogenic microorganisms and their persistence in the life cycle. Consequently, the contact between disinfectants and microorganisms can be challenging, as they may be internalized in the product, present on irregular surfaces, or in biofilms. Similarly, injuries caused by harvesting and transport can provide protected spaces where microorganisms can survive and grow, making disinfection difficult [138,139].

#### 2.4.1. Chlorine Disinfection 

Currently, the predominant disinfection methods involve the application of chlorine-based disinfectants [4,43]. However, these disinfectants pose risks to human health by generating carcinogenic compounds [7] and are not highly effective, as their disinfecting impact diminishes rapidly, allowing surviving bacterial populations to multiply more rapidly than those in non-disinfected products [43,46]. Chlorinated water is commonly utilized for disinfecting MPFV due to its cost-effectiveness and ease of use [43]. The efficacy of decontamination is assessed not only by the reduction achieved but, more crucially, by the ability to sustain this reduction over the product’s shelf life. Nevertheless, the use of active chlorine raises health concerns due to the formation of toxic by-products such as trihalomethanes and chloramines. This concern has led to restrictions on chlorine’s usage in several European countries, including the Netherlands, Sweden, Germany, Switzerland, Denmark, and Belgium [7,89]. A study by Coroneo et al. [147] investigated the presence of these derivatives during the disinfection process, concluding that toxic or carcinogenic compounds, specifically trihalomethanes, are formed and persist in the final product.

#### 2.4.2. Other Chemical Methods of Disinfection

Recent advancements have introduced various methodologies relying on chemical disinfectants, including chlorine dioxide [4,7,44], organic acids [4,7,43], hydrogen peroxide [7,40,44], electrolyzed water [4,7,43,44], ozonated water [4,7,43,44], and calcium-based solutions [7,148]. These methods have demonstrated ease of application and a potent bactericidal effect. However, most of them come with certain drawbacks. For instance, the use of chlorine dioxide, while effective in reducing bacterial populations, has been found to impact some organoleptic characteristics. Another consideration is the significant reduction of the native microbial population, which, by decreasing competition for space and nutrients, may potentially result in a subsequent increase in the development of pathogenic microorganisms [89].

#### 2.4.3. Physical Methods of Disinfection

Recent developments have introduced physical treatments such as ionizing radiation [4,7,40], ultraviolet [4,7,43,149], infrared [4,7,150], modified atmosphere packaging [7,40], or combinations such as ultrasound with ε-polylysine [151], aimed at preserving these types of products. Modified atmosphere packaging is a technique currently employed in the industry. These methods may be either bacteriostatic or bactericidal, demonstrating high efficiency in inhibiting microbial contaminations [85]. However, they come with certain challenges; for example, irradiation cannot be used as an isolated step of continuous washing, as it alone does not remove chemical residues or soil [7,44].

Table 8 summarizes the elimination methods referred to above, including major advantages and disadvantages.

### 2.5. Possible Future Solutions: The Use of Natural Disinfectants and Smart Packaging as an Alternative for Decontamination of Minimally Processed Fruits and Vegetables

As consumer preferences shift towards natural and minimally processed products with fewer chemical additives and extended shelf life, the use of synthetic antimicrobials is becoming more restricted due to potential toxicity concerns. Consequently, there is a growing need to identify alternative antibacterial substances, preferably of biological origin, that are both effective and harmless to human health and the environment. Natural antibacterial compounds have emerged as a promising alternative, gaining increased interest in their potential to eliminate pathogenic microorganisms, especially considering their resistance to antibiotics [152,153,154,155,156]. 

Numerous studies have explored new disinfection methods with the dual purpose of eliminating pathogens and preventing the degradation of vegetable products [44,155,157]. It is crucial to investigate techniques that not only decontaminate the product but also maintain low levels of microbiota over its shelf life. These compounds are derived from various sources, including plants (essential oils), microorganisms (such as lactic acid bacteria producing both lactic acid and antimicrobial polypeptides), and animals (for example, lysozyme) [153,157,158]. Antibacterial bioactive compounds are biological substances produced as defense against other organisms, and since these natural products and their components are generally recognized as safe (GRAS), concerns about their safety in preventing the development of pathogenic microorganisms or product alteration are minimal.

In recent decades, alternative compounds with the potential for food disinfection have emerged, including acetic acid, ascorbic acid, lactic acid, essential oils, and cheese whey, among others [152,158], all of which have less reported secondary effects and are more biodegradable. Smart packaging, an emerging technology in the food packaging industry, integrates active and intelligent features to enhance food safety and quality [159,160]. Over the past decades, innovative applications have surfaced, including the use of bioactive compounds such as essential oils (EO) in various packaging forms such as coating, nanoencapsulation, and synergistic pairings with other antibacterial agents [145]. Additionally, using materials with smart packaging properties, such as being impermeable to oxygen, light, moisture, and certain gases, contributes to minimizing spoilage by reducing microbial activity, with nanocomposite materials providing added resistance [159,160].

To optimize smart packaging development, it is crucial to consider cultural, social, and cognitive factors influencing consumer acceptance [161]. Tailoring these technologies to meet consumer preferences and needs, along with effective communication addressing consumer concerns and educating them on the benefits, will be key for successful implementation. However, despite these advancements, there are still limited natural disinfectants proposed in scientific studies that have reached the market. Traditional chemical methods, such as chlorinated compounds, continue to be used, emphasizing the importance of developing natural disinfectant products that can effectively replace chlorine-based products without compromising safety, environmental impact, or the organoleptic characteristics of the product.

## 3. One Health Perspectives on Food Safety in Minimally Processed Vegetables and Fruits

In today’s global food market, heightened consumer expectations for safe, high-quality, and affordable products underscore the importance of food safety in public health [162,163,164]. Paired with the need to respond to significant incidents such as Bovine Spongiform Encephalopathy (“mad cow disease”) and others, a global, more comprehensive approach has been followed, focusing mainly on risk assessment and the implementation of new regulations that mandate traceability, hygiene, hazard analysis critical control points (HACCP), and the withdrawal of unsafe products from the market [162,163].

However, in the era of globalization, food supply chains traverse multiple national borders, leading to the internationalization and amplification of health risks [163,164,165]. Despite earnest efforts, the challenge to food safety has never been more pronounced, with pathogen contamination giving rise to over 200 diseases, ranging from diarrhea to cancers [20]. Thus, it is becoming more and more clear that conventional approaches to guaranteeing food safety are inadequate, especially as our food systems becoming more complex [24,166]. Globalization has made food supply chains more complicated, which highlights the need for a comprehensive strategy to stop the spread of antibiotic resistance and microbial pathogens in processed foods. This shared responsibility acknowledges the interdependence of the entire food chain and goes from agricultural production to consumption. The food industry faces difficulties such as lengthier supply chains that result in longer transit times and quality risks because it works within a complex global supply chain. It is becoming more and more clear that to address these problems more comprehensively, foodborne outbreaks should be investigated cooperatively by the environmental, animal, and human health sectors, with a focus on One Health principles. This approach acknowledges the interconnectedness of health systems and emphasizes cross-sectoral collaboration. The lack of collaboration across the complete food production chain has hindered the identification of contamination sources and critical stages in the chain. Bridging “farm to fork” through a One Health approach, especially utilizing genomics, should be pursued to address this gap by comprehensively linking animal, food, environment, and human aspects in food production chains.

## 4. Conclusions

Ensuring the safety of food is fundamental for promoting healthy diets and sustainable food systems, aligning with the highest standards of nutrition and sustainability. It is crucial for food to be not only accessible and affordable but also free from hazards. As previously mentioned, a significant number of foodborne outbreaks are associated with the consumption of produce. These outbreaks are a result of societal changes, including shifts in agricultural practices, increased consumption of raw or minimally processed fruits and vegetables, a rise in immunocompromised consumers, and alterations in the distribution and trade of such products [7,40,74,134]. Addressing these outbreaks requires comprehensive control measures across the entire food chain, with a particular focus on primary production and processing. This becomes even more critical with the growing challenge of feeding an expanding global population, projected to reach 9.7 billion people by 2050, while maintaining food safety and health standards without exacerbating the environmental impact of food production and consumption [166].

To successfully achieve food safety and security, adopting a One Health approach is essential. It involves understanding and addressing the socio-economic contexts of food operators throughout the entire food chain, including consumers. The challenges in this regard will continue to intensify with increasing consumption patterns and the need for sustainable food solutions [166].

In the pursuit of enhanced food safety, institutions and individuals responsible for food safety should stay abreast of the latest developments in science and technology. Investing in future preparedness and embracing the One Health approach are key strategies. Research on alternative disinfectants, innovative food processing models, and emerging topics such as the microbiome hold significant importance. This knowledge is expected to be seamlessly integrated into future food safety assessments. Ultimately, the future of food safety in fresh produce hinges on holistic approaches that prioritize both health and safety, delivering natural and wholesome food products.

## Figures and Tables

**Table 1:** Main factors involved in the changing epidemiology of produce-associated foodborne illness; source: [52,56].

Changes in Industrial Production	Changes in the Consumption Habits	Others
Increasingly broad and centralized production	Ongoing trend toward greater consumption of foods not prepared at home	Increased proportion of vulnerable population (elderly, immunocompromised, chronic patients)
Distribution of products over large distances	Increase in salad bar popularity	Improved epidemiological surveillance
Increasing popularity of minimal processed products	Growing consumption of fresh fruits, vegetables, and natural juices	Enhance diagnostic and pathogens identification and traceability
Growth in global trade of fresh produce all over the world	Growing interest for healthier diets	Emerging pathogens with new skills and low infectious doses

**Table 2:** Mesophilic aerobic microorganism levels found in different types of minimally processed vegetables.

Fresh Cut Vegetables	Mesophilic Aerobic Microorganisms (log cfu.g^−1^)	Reference
Salads	5.5–7.4	[90]
Vegetables	5.3–7.5	[90]
Salads	3.0–6.6	[91]
Lettuce	4.57–6.78	[92]
Vegetables	5.47–7.82	[92]
Salads	3.8–9.4	[93]
Iceberg lettuce	6.03–8.43	[94]
Salads	5.8–7.1	[95]
Salads	2.36–9.30	[96]
Romaine Lettuce	5.71–7.89	[97]

**Table 3:** *Enterobacteriaceae*/coliform counts and percentage of positive samples for *Escherichia coli* (percentage higher than 2 log cfu.g ^−1^) in different types of minimally processed vegetables.

Fresh Cut Vegetables	*Enterobacteriaceae* (E) or Coliforms (C) log ufc.g^−1^	*Escherichia coli* % of Positive	References
Salads	1.9–6.0 (C)	30.0	[90]
Vegetables	<1.0–> 5.5 (C)	9.4	[90]
Salads	<0.48–3.1 (C)	-	[91]
Salads	-	10.0	[104]
Lettuce	2.18–5.66 (C)	25.7	[92]
Vegetables	0.48–> 5.04 (C)	16.0	[92]
Salads	4.15–6.15 (C)	4	[93]
Iceberg lettuce	-	0	[94]
Salads	4.4–6.9 (E)	6.7	[95]
Salads	1.30–7.48 (E)	50	[96]

**Table 4:** Overview of the presence of the three most important pathogens in ready-to-eat fruits and vegetables, juices, salads, spices and herbs, and sprouted seeds samples—data from Europe; source: [23].

RTE * Product	Microorganisms					
	*Salmonella* spp.		*L. monocytogenes*		STEC **	
	N° of Samples	% +	N° of Samples	% +	N° of Samples	% +
Fruit, vegetables, and juices	6261	0.05	1383	3.0	1922	0.52
Salads	2194	0.05	844	0.95	301	0
Spices and herbs	1529	0.72	115	0	296	0.34
Sprouted seeds	512	0	-	-	617	0.16

* Ready-to-eat; ** Shiga toxin-producing *E coli*.

**Table 5:** Estimated percentage of foodborne illnesses caused by *Salmonella*, *Escherichia coli* O157, and *Listeria monocytogenes* for 2020, based on multi-year outbreak data [119].

Product	Microorganisms		
	*Salmonella* spp.	*E. coli* O157	*L. monocytogenes*
Fruits	14.9	3.2	24.8
Seeded vegetables	12.0	1.2	-
Other produce	8.6	2.6	12.3
Vegetable row crops	4.1	58.1	14.1
Sprouts	3.7	1.5	2.9
Grains—beans	0.9	0.9	-
Total	44.2	67.5	54.1

**Table 6:** Foodborne outbreaks occurring in the United States of America linked to produce from 2018 to October 2023 [120].

Year	Product	Microorganism	Involved States	No. Cases	No. Hospitaliz.	No. Deaths
2018	Frozen Shredded Coconut	*Salmonella* I 4,[5],12:b:- and *Salmonella* Newport	9	27	6	0
	Raw Sprouts	*Salmonella* Montevideo	3	10	0	0
	Dried Coconut	*Salmonella* Typhimurium	8	14	3	0
	Romaine Lettuce	*Escherichia coli* O157:H7	36	210	96	5
	Pre-Cut Melon	*Salmonella* Adelaide	9	77	36	0
	Fresh Produce Vegetable Trays	*Cyclospora*	4	250	8	0
	Fresh Express Salad Mix	*Cyclospora*	16	511	24	0
	Romaine Lettuce	*Escherichia coli* O157:H7	16	62	25	0
2019	Pre-Cut Melons	*Salmonella* Carrau	10	137	38	0
	Fresh Papayas	*Salmonella* Uganda	9	81	27	0
	Fresh Basil	*Cyclospora*	11	241	6	0
	Romaine Lettuce	*Escherichia coli* O157:H7	27	167	85	0
	Salad Kits	*Escherichia coli* O157:H7	5	10	4	0
	Cut Fruit	*Salmonella* Javiana	14	165	73	0
2020	Clover Sprouts	*Escherichia coli* O103	10	51	3	0
	Enoki Mushrooms	*Listeria monocytogenes*	17	36	31	4
	Bagged Salad Mix	*Cyclospora*	14	701	38	0
	Onions	*Salmonella* Newport	48	1127	167	0
	Peaches	*Salmonella* Enteritidis	17	101	28	0
	Wood Ear Mushrooms	*Salmonella* Stanley	12	55	6	0
	Leafy Greens	*Escherichia coli* O157:H7	19	40	20	0
2021	Packaged Salad Greens	*Salmonella* Typhimurium	4	31	4	0
	Onions	*Salmonella* Oranienburg	39	1040	260	0
	Baby Spinach	*Escherichia coli* O157:H7	10	15	4	0
	Packaged Salads	*Listeria monocytogenes*	13	18	16	3
	Packaged Salads	*Listeria monocytogenes*	8	10	10	1
	Packaged Salads	*Escherichia coli* O157:H7	4	10	4	1
2022	Strawberries	Hepatitis A Virus	4	19	13	0
	Frozen Falafel	*Escherichia coli* O121	6	24	6	0
	Alfalfa Sprouts	*Salmonella* Typhimurium	8	63	10	0
2023	Leafy Greens	*Listeria monocytogenes*	16	19	18	0
	Frozen Strawberries	Hepatitis A	4	10	4	0

**Table 7:** Prevalence of *Clostridioides difficile* in produce.

Country	Product	% Positive	Reference
Scotland	Ready-to-Eat Salads	7.5% (3/40)	[132]
Canada	Vegetables	4.5% (5/111)	[122]
France	Ready-to-Eat Salads and Raw Vegetables	3.3% (3/104)	[128]
USA	Vegetable Products	2.4% (3/125)	[133]
Iran	Ready-to-Eat Salads	6% (6/106)	[131]
Australia	Root Vegetables	10% (30/300)	[124]
Japan	Fresh Vegetables	3.3% (8/242)	[125]

**Table 8:** Main decontamination methodologies used in the industry for minimally processed fruits and vegetables and respective advantages and disadvantages.

Disinfection Method	Effect	Advantages	Disadvantages	References
Chlorine	Antimicrobial efficacy related to oxidation capacity by a short contact time in chilled water	Ease and economic application	High amount of highly polluted wastewater Occurrence of residues of trihalomethanes and chloramines Banned in some European countries	[7,43]
Chlorine dioxide	Antimicrobial efficacy related to higher oxidative capacity than chlorine	No reaction with nitrogen-containing compounds to form carcinogenic by-products	Requires long exposure time, which affects the organoleptic properties of the product	[4,7,44]
Organic acids	Reduction in internal cellular pH, disruption of membrane transport and permeability Anion accumulation	Washes of lactic, citric, acetic, tartaric, and ascorbic acid rapidly inactivate a broad spectrum of bacteria Maintain the products’ quality Considered GRAS	Low antimicrobial efficacy	[4,7,43]
Hydrogen peroxide	Strong oxidizing power Generates other cytotoxic oxidizing species such as hydroxyl radicals	Excellent disinfectant agent mostly employed into post-harvest facilities (spaces and materials) Used in preventing post-harvest losses in table grapes, potatoes, strawberries, and lemons rather than in disinfecting fresh and MPFV produce	Reactive oxygen species being toxic to living cells Causes browning of shredded lettuce	[7,44]
Ozonated water	Strong oxidative and microbial agent	Allows extended shelf life High reactivity Spontaneously decomposes to oxygen, leaving no residues on treated produce Active against bacteria, fungi, virus, and bacterial and fungal spores Considered GRAS	Long exposure time is needed Corrosiveness of products Capital cost	[4,7,43,44]
Electrolyzed water (EW)	Has a strong bactericidal effect	Low operational expenses Easy operation Safe and eco-friendly No significant alteration of product quality Reduced concentration of chlorine in the wash water	High cost and limited availability of equipment Very short shelf life and requires on-site generation Corrosive hazard of strong acid EW Cl_2_ production	[4,7,44]
Calcium-based solutions	Maintain the vegetable cell wall integrity Inhibit plant tissue senescence Have antibacterial properties Calcium salts have been used as a firming agent for fruit	Allows extended shelf life The final product can significantly increase the calcium content	Limited efficacy as antimicrobial Bitterness and off flavors associated with calcium chloride May be too expensive	[7]
Ionizing radiation	Reduces bacteria, yeast, molds, parasites, protozoa, and insects Inactivates genetic material of the living cells	Environmentally friendly and time effective Ionizing irradiation shows beneficial effects in reducing the microbial population Extend the shelf life and maintain the quality of fruits and vegetables. Leaves no residue on the food	A high-dose irradiation is required Quality may be affected Texture alteration	[4,7,40]
Ultraviolet	Strong antibacterial agent due to genetic damage Induction of resistance mechanisms in different fruit and vegetables against pathogens	Relatively inexpensive and easy-to-use equipment Broad-spectrum bactericidal effect Allows extended shelf life Environmentally friendly	Can cause damage to the treated tissue and increased stress and respiration rate Induces a lignifications-like process Low penetration of UV light Long treatment times Negative impact on product sensory quality Low efficacy at high organic matter level Complex standardization of application at commercial scale	[4,7,43,149]
Modified atmosphere packaging (MAP)	Low levels of O_2_ and high levels of CO_2_ reduce the produce respiration rate, with the benefit of delaying senescence	Allows extended shelf life Preserves quality Fresh-cut products are more tolerant to higher CO_2_ concentrations than intact products	Changes of the gas composition Fermentation and formation of off flavor compounds May allow growth of pathogenic bacteria	[7,40]

## Data Availability

Not applicable.
